# Molecular evolution of enzymes involved in the arachidonic acid cascade

**DOI:** 10.1155/S0962935192000346

**Published:** 1992

**Authors:** Hiroyuki Toh, Yoshihiro Urade, Tadashi Tanabe

**Affiliations:** 1 Protein Engineering Research Institute 6-2-3 Furuedai Suita 565 Japan; 2 International Research Laboratories CIBA-GEIGY (Japan) 10-66 Miyuki-Cho Takarazuka 665 Japan; 3 National Cardiovascular Center Research Institute 5-7-1 Fujisiro-dai Suita 565 Japan

## Abstract

The metabolic pathway called the arachidonic acid cascade produces a wide range of eicosanoids, such as prostaglandins, thromboxanes and leukotrienes with potent biological activities. Recombinant DNA techniques have made it possible to determine the nucleotide sequences of cDNAs and/or genomic structures for the enzymes involved in the pathway. Sequence comparison analyses of the accumulated sequence data have brought great insights into the structure, function and molecular evolution of the enzymes. This paper reviews the sequence comparison analyses of the enzymes involved in the arachidonic acid cascade.


**Invited Review**

Mediators of Inflammation 1, 223–233 (1992)

### Molecular evolution of enzymes involved in the arachidonic acid cascade

**Hiroyuki Toh,[1,CA] Yoshihiro Urade[2] and Tadashi Tanabe[3]**

[1]Protein Engineering Research Institute, 6-2-3, Furuedai, Suita 565, Japan; [2]International Research Laboratories, CIBA-GEIGY (Japan), 10-66, Miyuki-Cho, Takarazuka 665, Japan; and [3]National Cardiovascular Center Research Institute, 5-7-1, Fujisiro-dai, Suita 565, Japan

[CA] Corresponding Author

**The metabolic pathway called the arachidonic acid cascade produces a wide range of eicosanoids, such as prostaglandins, thromboxanes and leukotrienes with potent biological activities. Recombinant DNA techniques have made it possible to determine the nucleotide sequences of cDNAs and/or genomic structures for the enzymes involved in the pathway. Sequence comparison analyses of the accumulated sequence data have brought great insights into the structure, function and molecular evolution of the enzymes. This paper reviews the sequence comparison analyses of the enzymes involved in the arachidonic acid cascade.**

**Key words:** Arachidonic acid cascade, Brain-type PGD synthase, Cyclooxygenase, Lipoxygenases, LTA hydrolase, PGF synthase, TXA synthase

### Introduction

Arachidonic acid is released from cell membranes by chemical, mechanical or electrical stimuli. The deesterified arachidonic acid is oxygenated to yield a variety of bioactive compounds—prostaglandins (PGs), thromboxanes (TXs), leukotrienes (LTs), and lipoxins (LXs). These oxygenated arachidonic acid compounds are collectively termed 'eicosanoids'. Most eicosanoids act as critical mediators in inflammation, blood clotting, control of vascular tone, renal function, reproductive systems, and cell differentiation. In addition, neuronal and neuroendocrine functions of eicosanoids have been discovered recently, and it has been indicated that eicosanoids play more important roles in intercellular signal transduction systems than those considered previously. The metabolic pathway where eicosanoids are formed, is called the 'arachidonic acid cascade' (see References 1, 2, 3 and 4 for reviews).

One of the prominent achievements in the research of the arachidonic acid cascade is molecular cloning of the cDNAs and/or genes for the key proteins involved in the cascade.[3,4] These proteins include not only the synthases of eicosanoids, but also a receptor for an eicosanoid[5] and an activation factor for a synthase.[6] Sequence comparison analyses of the sequence data obtained have given clues to the structures and functions of the proteins. We focus here on the synthases of eicosanoids, and discuss the structure, function and molecular evolution of these enzymes based on the sequence comparison analyses.

### Cyclooxygenase

The arachidonic acid cascade consists of four metabolic pathways; cyclooxygenase pathway, lipoxygenase pathway, P-450 pathway and autooxidation pathway.[3] Cyclooxygenase (PG endoperoxide synthase, PGG/H synthase) catalyses the first step in the cyclooxygenase pathway, in which PGs, TXs and prostacyclin are produced. Cyclooxygenase is a bifunctional enzyme with cyclooxygenase activity (arachidonic acid to $PGG_2$) and peroxidase activity ($PGG_2$ to $PGH_2$),[7,8] and iron(III)-protoporphyrin IX is required for both activities.[7,9,10] The nucleotide sequences of cDNAs for this enzyme derived from sheep,[11–13] mouse[14] and human[15] have been determined. In addition, the genomic structure for human cyclooxygenase has been determined.[16] The deduced amino acid sequences are about 600 residues in length, which include a signal sequence of about 24 residues. They are referred to here as cyclooxygenases-1.

Recently, it was found that a mRNA induced by Rous sarcoma virus in chicken embryo fibroblasts encodes a protein homologous to cyclooxygenase-1.[17] Furthermore, a mRNA induced by phorbol ester in Swiss3T3 cells was reported to encode a cyclooxygenase homologue.[18] The genomic structure of the latter was recently determined.[19] These are referred to here as cyclooxygenases-2, although the details of their functions have not been established.

The C-terminal region of this enzyme of about 400 residues is homologous to the C-terminal region of a wide variety of peroxidases—myeloper-

© 1992 Rapid Communications of Oxford Ltd

**a**

```

       1 .........+.........+.........+.........+.........+.........+...

                          *

(1)    GQEVFGLLPGLMLYATIWLREHNRVCDLLKAEHPTWGDEQLFQTARLILIGETIKIV-IEEYV

(2)    GQEVFGLLPGLMLYATLWLREHNRVCDLLKAEHPTWGDEQLFQTTRLILIGETIKIV-IEEYV

(3)    GQEVFGLLPGLMLFSTIWLREHNRVCDLLKEEHPTWDDEQLFQTTRLILIGETIKIV-IEEYV

(4)    GQEVFGLVPGLMMYATIWLREHNRVCDVLKQEHPEWDDEQLFQTTRLILIGETIKIV-IEDYV

(5)    GQEVFGLVPGLMMYATIWLREHNRVCDILKQEHPEWGDEQLFQTSRLILIGETIKIV-IEDYV

(6)    GDGRASEVPALAAVHTLWLREHNRLATAFKAINTHWSANTAYQEARKV-VGALHQIITMRDYI

(7)    GDGRASEVPSLTALHTLWLREHNRLAAALKALNAHWSADAVYQEARKV-VGALHQIITLRDYI

(8)    GDSRASEVPGLTALHTLWLREHNRLAAAFKALNAHWSADTVYQEARKV-VGALHQIVTLRDYV

(9)    GDFRASEQILLATAHTLLLREHNRLARELKKLNPHWNGEKLYQEARKI-LGAFIQIITFRDYL

(10)   GDMRSSEMPELTSMHTLFVREHNRLATQLKRLNPRWNGEKLYQEARKI-VGAMVQIITYRDYL

(11)   GDTRSSEMPELTSMHTLLLREHNRLATELKSLNPRWDGERLYQEARKI-VGAMVQIITYRDYL

(12)   GDTRSTETPKLAAMHTLFMREHNRLATELRRLNPRWNGDKLYNEARKI-MGAMVQIITYRDFL

       ●○   ○    ●    ●○ ○●●●●●●○     ○    ○ ●  ○  ○○ ○● ○ ○●      ●○   ○○○

```

**b**

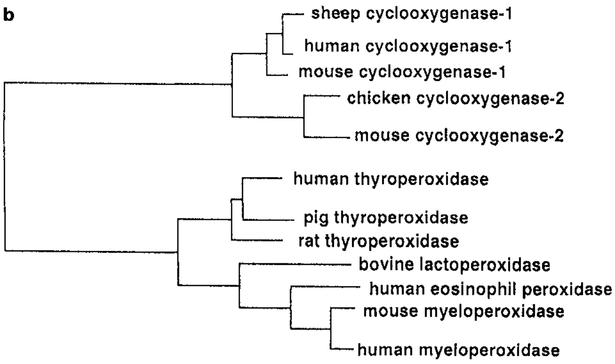

**c**

```
       1 .........+.........+.........+.........+.........+.....
(1)    PVNPCC---YYP-CQHQGICVRF-GLDRYQCDCTRTGYSGPNCTIPEI-WTW-LR
(2)    PVNPCC---YYP-CQHQGICVRF-GLDRYQCDCTRTGYSGPNCTIPGL-WTW-LR
(3)    PVNPCC---YYP-CQNQGVCVRF-GLDNYQCDCTRTGYSGPNCTIPEI-WTW-LR
(4)    --NPCC---SLP-CQNRGVCMTT-GFDRYECDCTRTGYYGENCTTPEF-FTW-LK
(5)    --NPCC---SNP-CQNRGECMST-GFDQYKCDCTRTGFYGENCTTPEF-LTR-IK
(6)    NSYPGCPSSYDGYCLNGGVCMHIESLDSYTCNCV-IGYSGDRCQTRDLRW-WELR
(7)    NSNTGCPPSYDGYCLNGGVCMYVESVDRYVCNCV-IGYIGERCQHRDLR-----
(8)    NSDSECPLSHDGYCLHDGVCMYIEALDKYACNCV-VGYIGERCQYRDLKW-WELR
           ○ ●     ○ ●   ● ●○   ○ ● ● ●○●   ●○ ●  ●
```

FIG. 1. (a) Alignment of the peroxidase family around an invariant His residue. Redrawn from the alignment in Ref. 20. Asterisk indicates the invariant His residue. '-' indicates gap. Open and closed circles under the alignment indicate invarant sites and sites occupied by physicochemically similar amino acid residues. (1) Sheep cyclooxygenase-1,[11] (2) Human cyclooxygenase-1,[16] (3) Mouse cyclooxygenase-1,[14] (4) Chicken cyclooxygenase-2,[17] (5) Mouse cyclooxygenase-2,[18] (6) Rat thyroid peroxidase (S07047), (7) Human thyroid peroxidase (A32413), (8) Pig thyroid peroxidase (A27416), (9) Bovine lactoperoxidase (S16103), (10) Mouse myeloperoxidase,[92] (11) Human myeloperoxidase,[93] and (12) Human eosinophil peroxidase (A34408). The codes in the parentheses indicate the accession numbers of the proteins in the NBRF database as references.

(b) Phylogenetic tree of the peroxidase family. Redrawn from the tree in Ref. 20.

(c) Alignment of EGF-like domains of cyclooxygenases and EGFs. EGF-like domains of (1) Sheep cyclooxygenase-1,[11] (2) Human cyclooxygenase-1,[16] (3) Mouse cyclooxygenase-1,[14] (4) Chicken cyclooxygenase-2,[17] and (5) Mouse cyclooxygenase-2,[18] and EGFs of (6) Mouse (EGMSMG), (7) Rat (EGRT), and (8) Human (EGHU). The codes in the parentheses indicate the accession numbers of the proteins in the NBRF data base as references. Open circles, closed circles and '-' have the same meanings as those in Fig. 1(a).

oxidase, eosinophil peroxidase, thyroid peroxidase and lactoperoxidase.[12,20] In particular, the region around an invariant His residue (residue position 309 in sheep cyclooxygenase) is remarkably conserved (Fig. 1(a)).[14,20] The invariant His residue is considered to act as a ligand for heme. Figure 1(b) shows a phylogenetic tree of these enzymes,[20] which indicates the early divergence of cyclooxygenases from the other peroxidases. The cluster made by cyclooxygenases-1 is distinct from that made by cyclooxygenases-2. Considering that cyclooxygenases-2 are derived from chicken and mouse, the divergence between the genes of cyclooxygenases-1 and -2 occurred before the species divergence between avians and mammals. This observation suggests that mammals and avians have at least two copies of cyclooxygenase-related genes on their genomes. On the other lineage of the peroxidase family, thyroid peroxidase functionally diverged first. Then, lactoperoxidase diverged. The divergence between myeloperoxidase and eosinophil peroxidase occurred last.[20]

Both cyclooxygenases-1[21] and -2[20] contain an epidermal growth factor (EGF)-like domain downstream of the signal sequences (Fig. 1(c)). The EGF-like domains have been found in a wide variety of proteins,[22] and are considered to have been introduced into those proteins by exon shuffling.[23] The EGF-like domain is encoded by the third exon in the human cyclooxygenase gene.[16] The cyclooxygenase has been shown immunohistochemically to be localized at the endoplasmic reticulum and the nuclear membrane in Swiss 3T3 cells.[24]

Considering that EGF-like domains contain disulphide bridges, the region containing the EGF-like domain of the cyclooxygenase may at least be present at the luminal surface. However, EGF and EGF-like domains are found ordinarily in extracellular regions, and only the EGF-like domain of thyroid peroxidase was reported to be present in the luminal surface of endoplasmic reticulum.[25] Therefore, the EGF-like domain of this enzyme is considered to be a second example of the domain found in cells. However, the functional meaning of this domain in this enzyme has not yet been established which is true for most of the EGF-like domains. Cyclooxygenase contains the EGF-like domain at its N-terminal region, while thyroid peroxidase contains the EGF-like domain at its C-terminal region. Therefore, it is considered that exon shuffling had occurred independently twice, in order to introduce EGF-like domains into the members of the peroxidase family during the course of evolution.[21]

### Brain-type PGD Synthase

$PGD_2$ is a major PG produced in the brain of humans and rats, and shows a wide variety of activities *in vivo* such as sleep induction, hypothermia, anticonvulsion, nociception, suppression of luteinizing hormone release, and modulation of the odour response.[26,27] Brain-type PGD synthase catalyses the isomerization of $PGH_2$ to form $PGD_2$. The nucleotide sequences of cDNAs for the enzyme from rats[28] and humans[29] were determined. In addition, the genomic structure for the rat enzyme was reported.[30] The deduced amino acid sequences are about 190 residues in length, including a signal sequence of 20 residues.

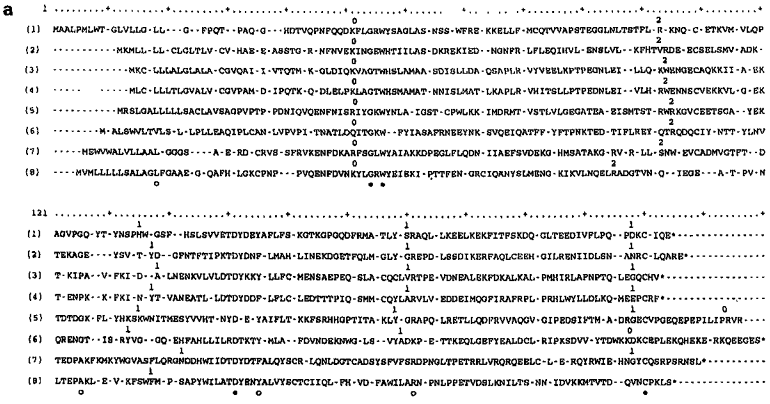

This enzyme shows weak (10–33% identity) but significant sequence homology to the members of the lipocalin family in their entire regions.[29,31] The lipocalin family consists of a wide variety of small secretory proteins (160–200 amino acids in length), which includes $\beta$-lactoglobulin, retinol-binding protein, bilin-binding protein, $\alpha_1$-acid glycoprotein, odorant-binding protein and $\alpha_2$-urinary globulin.[32,33] These proteins are bound to small lipophilic molecules and are involved in the transport of those molecules. The amino acid sequences of the members are different from one another, but six conserved regions are identified, which characterize the lipocalins (alignment positions 51, 54–56, 109, 150–152, 181 and 220 in Fig. 2(a)).[32] All these a b

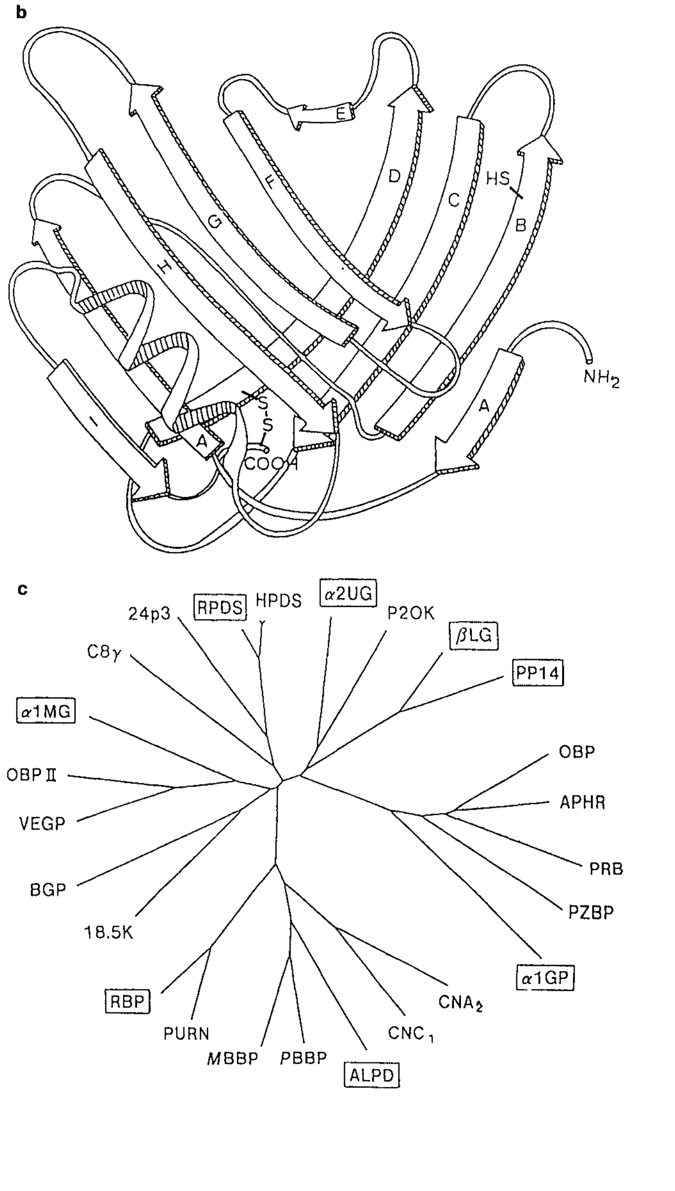

FIG. 2. (a) Alignment of rat brain-type PGD synthase and seven lipocalins whose genomic structures are known. Redrawn from the alignment in Ref. 30. Asterisks indicate stop codons. The positions of the exon/intron junction are indicated by the numbers on the sequences. '0' between two residues indicates splicing between codons. '1' and '2' on a residue indicate splicing between the first and second positions of a codon, and that between the second and third positions of a codon, respectively. Closed circles, open circles, and '-' have the same meanings as those in Fig. 1(a). (1) Rat brain-type PGD synthase,[30] (2) Mouse $\alpha_2$-urinary globulin,[94] (3) Ovine $\beta$-lactoglogulin,[32] (4) Human placental protein 14 (A35570), (5) Human $\alpha_1$-microglobulin,[95] (6) Human $\alpha_1$-acid glycoprotein,[96] (7) Rat retinol-binding protein (VART), and (8) Human apolipoprotein D (A26958). The codes in the parentheses indicate accession numbers in the NBRF data base as references.

(b) Model tertiary structure of PGD synthase. Redrawn from the model in Ref. 29.

(c) Unrooted phylogenetic tree of 26 lipocalins including brain-type PGD synthases derived from humans and rats. Re-drawn from the alignment in Ref. 30. Lipocalins whose tertiary structures are known are boxed. The abbreviations are as follows: RPDS, rat brain-type PGD synthase;[30] HPDS, human brain-type PGD synthases;[29] 24p3, mouse 24p3 protein (S07397); C8$\gamma$, human complement component C8$\gamma$ chain (C8HUG); $\alpha$2UG, rat $\alpha_2$-urinary globulin;[97] p20K, chicken quiescene-specific polypeptide 20K (A30230); $\beta$LG, ovine $\beta$-lactoglobulin;[32] PP14, human placental protein 14 (A31242); OBP, rat odorant-binding protein (A28713); APHR, hamster aphrodisin (A31243); PRB, rat probasin (A32602); PZBP, bovine pyrazine-binding protein (S06843); $\alpha$1GP, human $\alpha_1$-acid glycoprotein (OMHU1); $\alpha$1MG, human $\alpha_1$-microglobulin (HCHU); OBPII, rat odorant-binding protein II;[98] VEGP, rat von Ebner's gland protein (S08161); BGP, frog, *Rana pipiens*, Bowman's gland protein (OVFGP); 18.5K, rat androgen-dependent epididymal 18.5K protein (SQRTAD); RBP, rat retinol-binding protein (VART); PURN, chicken retinol-binding protein (A26969); *M*BBP, tobacco hornworm, *Manduca sexta*, bilin-binding protein (CUWIO); *P*BBP butterfly, *Pieris brasica*, bilin-binding protein (S00819); ALPD, human apolipoprotein D (A26958); $CNA_2$, lobster, *Homarus gammarus*, crustcyanin $A_2$.[99] $CNC_1$, lobster, *Homarus gammarus*, crustcyanin $C_1$.[100] The codes in the parentheses indicate the accession numbers of the proteins in the NBRF data base as references.

conserved regions are also found in the PGD synthases.[29] In addition to the similarity in sequence and size, the positions of exon/intron junctions and the phases of the splicing of this enzyme are similar to those of the lipocalins (see Fig. 2(a)).[30]

The fact that tertiary structures of proteins are more conserved than their primary structures has been amply documented.[34–36] In fact, three lipocalins, $\beta$-lactoglobulin,[37,38] retinol-binding protein[39,40] and bilin-binding protein,[41,42] share a common folding pattern called $\beta$-barrel structure in spite of the high sequence divergence among them (13–22% identity). The hydrophobic ligands are bound in the barrel structure. The sequence homology between PGD synthases and lipocalins suggests that the enzyme also has a $\beta$-barrel structure[29,31] (Fig. 2(b)). The enzyme is known to bind to several small lipophilic molecules including $PGH_2$, the substrate for this enzyme.[29] These molecules may be bound in the barrel structure as with other lipocalins.

However, this enzyme has two distinctive characteristics compared to other lipocalins.[29,31] One is that this enzyme has an enzymatic activity, whereas the other lipocalins are involved in the transport of small lipophilic molecules. Since the enzyme requires sulphydryl compounds for the reaction and is inactivated by treatment with sulphydryl modifiers, Cys residues are considered to be involved in the catalytic site of the enzyme. The sequence comparison between the enzymes from humans and rats revealed three conserved Cys residues. Two of them (alignment positions 109 and 220 in Fig. 2(a)) are also conserved in the other lipocalins, and are involved in the disulphide bridge formation. The remaining Cys residue is specifically conserved in the PGD synthase (alignment site 82 in Fig. 2(a)), which may play an important role in its enzymatic activity. The Cys residue of alignment site 82 is present in the predicted $\beta$-barrel structure favourable for interaction with its substrate (see Fig. 2(b)).

The other distinctive characteristic is that this enzyme is a membrane associated protein, whereas the other lipocalins, except for an isoform of probasin (which reportedly translocates to the nucleus[43]), are secretory proteins. It is an important and interesting problem not only for molecular evolution but also for protein engineering to establish the mechanism for the acquisition of the two characteristics.

Figure 2 (c) shows a phylogenetic tree of the 26 lipocalins including three PGD synthases.[30] The tree shows that PGD synthase is relatively close to the p23 oncogene product and the complement component C8 $\gamma$ chain, although the similarities of the enzyme to these two lipocalins are low (23–33% identity).

### PGF Synthase

$PGF_{2\alpha}$ shows activities of bronchoconstriction, vasoconstriction, luteolysis, and acetylcholine release. On the other hand, 11-*epi*-$PGF_{2\alpha}$, the isomer of $PGF_{2\alpha}$, causes bronchoconstriction, vasoconstriction, $Na^+$ excretion, urinary excretion, and antiaggregation. The former is converted from $PGH_2$ and the latter from $PGD_2$ by the same enzyme, PGF synthase. Although both activities of this enzyme require NADPH, the active site for the $PGF_{2\alpha}$ formation is different from that for 11-*epi*-$PGF_{2\alpha}$ formation.[44,45] The cDNAs encoding this enzyme were isolated from bovine lung[46] and liver.[47] Both cDNAs encode proteins of 323 amino acids which are closely related to each other (99% identity).

PGF synthase shows high sequence similarity to the members of the aldo-keto reductase family (36–74% identity).[46] In the presence of NADPH, PGF synthase catalyses the reduction of not only $PGD_2$ and $PGH_2$, but also several carbonyl compounds including 9,10-phenanthrenequinone, a substrate of the aldo-keto reductases.[48] On the other hand, human liver aldehyde reductase, whose sequence shows 40% identity to that of the PGF synthase, catalyses the reduction of $PGH_2$, although it does not catalyse the reduction of $PGD_2$.[49] The aldo-keto reductase family consists of a wide variety of reductases, such as human liver chlordechone reductase, *Corynebacterium* diketogluconic acid reductase, human liver aldehyde reductase, and aldose reductases from various mammals. Figure 3(a) is a multiple alignment of the family including the PGF synthases, and Fig. 3(b) shows an unrooted phylogenetic tree of the family. As shown in the figure, this enzyme shows the highest sequence similarity to chlordechone reductase within the members of the family (74% identity). It is interesting that frog $\rho$-crystallin occupies a position relatively close to PGF synthase and chlordechone reductase in the tree.[50,51] The amino acid sequence of frog $\rho$-crystallin shows 59% identity against that of PGF synthase or chlordechone reductase. Recently, it was revealed that the $\rho$-crystallin has an NADPH-binding activity and shows a weak enzymatic activity to convert $PGH_2$ to $PGF_{2\alpha}$ (5% of the activity of PGF synthase), although it does not show the activity to convert $PGD_2$ to 11-*epi*-$PGF_{2\alpha}$.[51]

There are several crystallins apart from the frog $\rho$-crystallin, which have been identified from various animals and show high sequence similarity to the other functionally important enzymes. For example, $\varepsilon$-crystallin from avian and crocodalian lenses shows high sequence similarity to the lactate dehydrogenase B4. Furthermore, the $\varepsilon$-crystallin shows an enzymatic activity comparable to the a

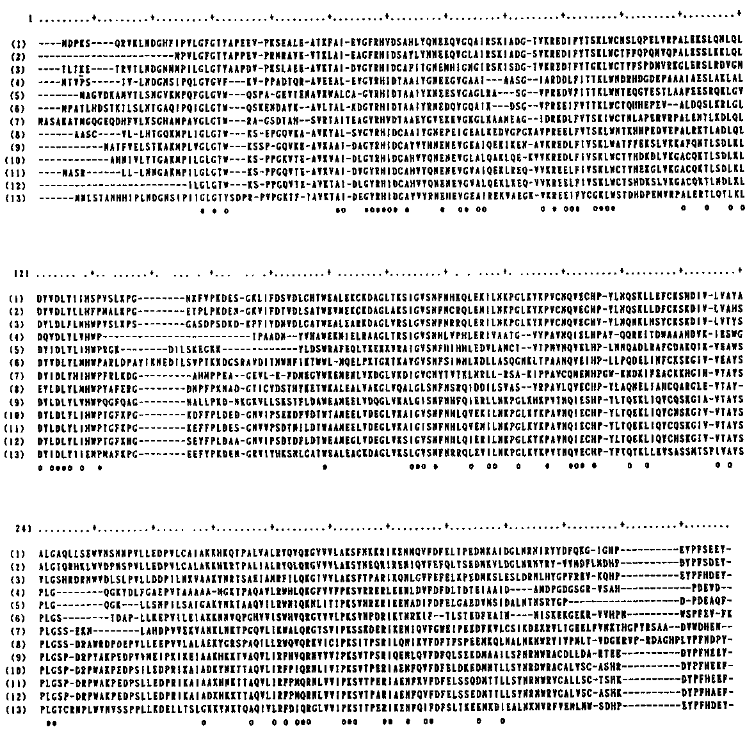

FIG. 3. (a) Alignment of aldo-keto reductase family including PGF synthases. Redrawn from the alignment in Ref. 51. Open circles, closed circles, and '-' have the same meanings as those in Fig. 1(a). (1) Bovine lung PGF synthase,[46] (2) Human chlordecone reductase (A34263), (3) Frog, *Rana temporaria*, *ρ*-crystallin,[51] (4) *Corynebacterium* diketogluconic acid reductase,[101] (5) *Leishmania major* P100/11E gene product (A32950), (6) Yeast nuclear gene GCY product,[102] (7) Barley aldose reductase-like protein (S15024), (8) Human aldehyde reductase,[103] (9) Mouse androgen-dependent protein (A37990), aldose reductases derived from (10) Bovine (A35452), (11) Human (S06591), and (12) Rabbit (A34406), and (13) Rat. $\Delta^4$-3-ketosteroid 5$\beta$-reductase (S15835). The codes in the parentheses indicate the accession numbers of the proteins in the NBRF data base as references.

(b) Unrooted phylogenetic tree of the aldo-keto reductase family. The nodes in a circle indicate proteins which belong to a group designated near the circle.

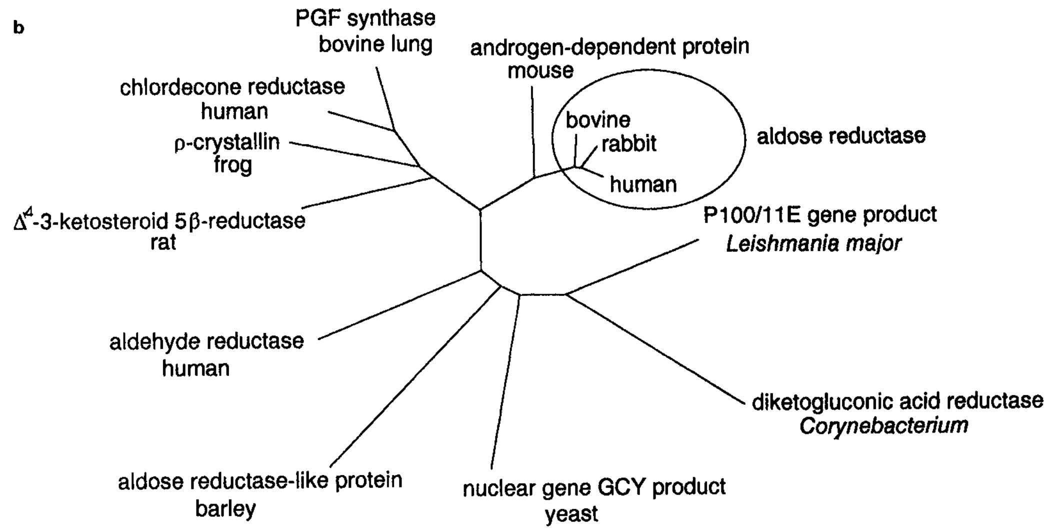

b homologous enzyme. Based on these observations, a new molecular evolutionary mechanism was proposed. The functional divergence of a gene product has been considerd to be accompanied by the duplication of the gene. That is, the relaxation of the selection pressure to the gene by gene duplication makes it possible for one copy to acquire a new function. However, the above observation suggests another evolutionary mechanism, that is, a gene can acquire new functions without loss of the original function or gene duplication. This new mechanism is called 'gene sharing'.[52] The sequence homology between PGF synthase and frog *ρ*-crystallin gives an example of gene sharing.

Recently, the crystal structure of pig aldose reductase was determined.[53] The enzyme exhibits a single domain protein with an eight-stranded parallel $\alpha/\beta$ barrel, or a so-called triose phosphate isomerase (TIM)-barrel structure. The crystallographic analysis revealed that 2′-monophosphoadenosine-5′-diphospho-ribose, which competitively inhibits the binding of NADPH, binds to a cleft located at the C-terminal region of the TIM-barrel structure. This observation suggests that PGF synthase also has a TIM-barrel structure and NADPH is bound to the C-terminal region of this enzyme.

### TXA Synthase

$TXA_2$ is a potent inducer of platelet aggregation and a constrictor of smooth muscles.[54,55] It is known that this eicosanoid is involved in several diseases such as thrombosis, atherosclerosis and asthma.[56] TXA synthase catalyses the conversion of $PGH_2$ to $TXA_2$. TXA synthase and PGI synthase were purified to homogeneity, and were found to be the cytochrome P-450 enzymes.[57,58] Recently, cDNA of this enzyme was identified from human platelet[59] and lung cDNA libraries,[60] and the complete sequence of the cDNA was reported. The cDNA encodes an amino acid sequence of 533 amino acids.

Cytochrome P-450s are ubiquitous in nature and are found in animals, plants, fungi and bacteria. P-450s comprise multigene families in living organisms, and evolutionary relationships among them have been investigated extensively.[61–63] TXA synthase (and PGI synthase) is involved in the cyclooxygenase pathway. Additionally, P-450s are involved in the cytochrome P-450 pathway to yield various epoxy-eicosatrienoic acids from arachidonic acid by their monooxygenase activities.[64] On the other hand, PG $\omega$-hydroxylase was cloned from rabbit lung,[65] and is involved in $\omega$-oxidation, a step in metabolic inactivation of PGs. PG $\omega$-hydroxylase also belongs to the cytochrome P-450 family.

Figure 4(a) shows the phylogenetic relationships between 50 members of the P-450 family including TXA synthase and PG $\omega$-hydroxylase.[66] PG $\omega$-hydroxylase belongs to the P-450 IV family. Contrary to that, TXA synthase occupies a unique position in the tree, but it is relatively close to the P-450 III family, as noted previously.[59] Figure 4(b) shows the alignment of the TXA synthase and cytochrome P-450 III. The Glu (alignment site 406), Arg (alignment site 409), Phe (alignment site 479), Cys (alignment site 486) residues are invariant in the 50 sequences used for construction of the phylogenetic tree.

The tertiary structure of P-450 CI (or P-450cam) has been determined.[67] Although the sequence identity of P-450 CI to other eukaryotic P-450s is very weak, the other P-450s are considered also to have a similar folding pattern to that of P-450 CI, and modellings of the tertiary structures of other P-450s according to the sequence homology were tried.[68] The sequence identity between P-450 CI and TXA synthase is also low, but it is considered that TXA synthase also has a tertiary structure like that of P-450 CI.

### 5- 12- and 15-Lipoxygenases

We have discussed the structure, function and molecular evolution of the enzymes involved in the cyclooxygenase pathway. Now we will discuss the enzymes involved in the lipoxygenase pathway, the other constituent of the arachidonic acid cascade. The lipoxygenase pathway is further divided into three pathways, each of which is initiated by the oxygenation of arachidonic acid by 5-, 12- and 15-lipoxygenases, respectively.[3,4] The numbers attached to the names of the enzymes indicate the carbon positions of the arachidonic acid, which are subjected to the oxygenation catalysed by the corresponding enzymes. The nucleotide sequences of the cDNAs for the three types of the lipoxygenases (5-lipoxygenases derived from humans[69,70] and rats;[71] 12-lipoxygenases derived from humans[72,73] and pigs;[74] and 15-lipoxygenases derived from humans[75] and rabbits[76]) have been determined. Furthermore, the genomic structures of human 5-lipoxygenase[77] and rabbit 15-lipoxygenases[78] were determined. The deduced amino acid sequences are about 700 residues in length, and the primary structures of 5-, 12- and 15-lipoxygenases are homologous to each other in their entire regions. Plants also contain several lipoxygenases of about 900 amino acid residues in length. The animal and plant lipoxygenases show sequence similarity at their C-terminal regions of about 500 amino acid residues. However, their N-terminal regions are different not only in amino acid sequences but also in the lengths.[20]

Sequence comparison of animal and plant lipoxygenases suggests that their primary structures contain a cluster of invariant His residues (Fig. 5(a)). It is known that lipoxygenases contain a non-heme iron co-factor, and the invariant His residues were proposed to act as ligands for the iron co-factor.[75,79] On the other hand, animal 12- and 15-lipoxygenases were reported to contain a zinc finger-like motif, which was also proposed to act as a ligand for the iron co-factor. However, the motif is absent in animal 5-lipoxygenases and plant lipoxygenases (Fig. 5(b)).[72,74]

Figure 5(c) shows a phylogenetic tree of the lipoxygenases.[20] Two hypothetical ancestral lipoxygenases are shown in the tree (lipoxygenase-X and lipoxygenase-Y). Animal and plant lipoxygenases diverged at node 'a'. Then, 5-lipoxygenase and lipoxygenase-X diverged by gene duplication at node 'b'. Next, human 12-lipoxygenase and lipoxygenase-Y functionally diverged at node 'c' by gene duplication of lipoxygenase-X. After that, pig 12-lipoxygenase and 15-lipoxygenase diverged at node 'd' by gene duplication of lipoxygenase-Y. Hence, the tree suggests that enzymes with 12-lipoxygenase activity were created independently

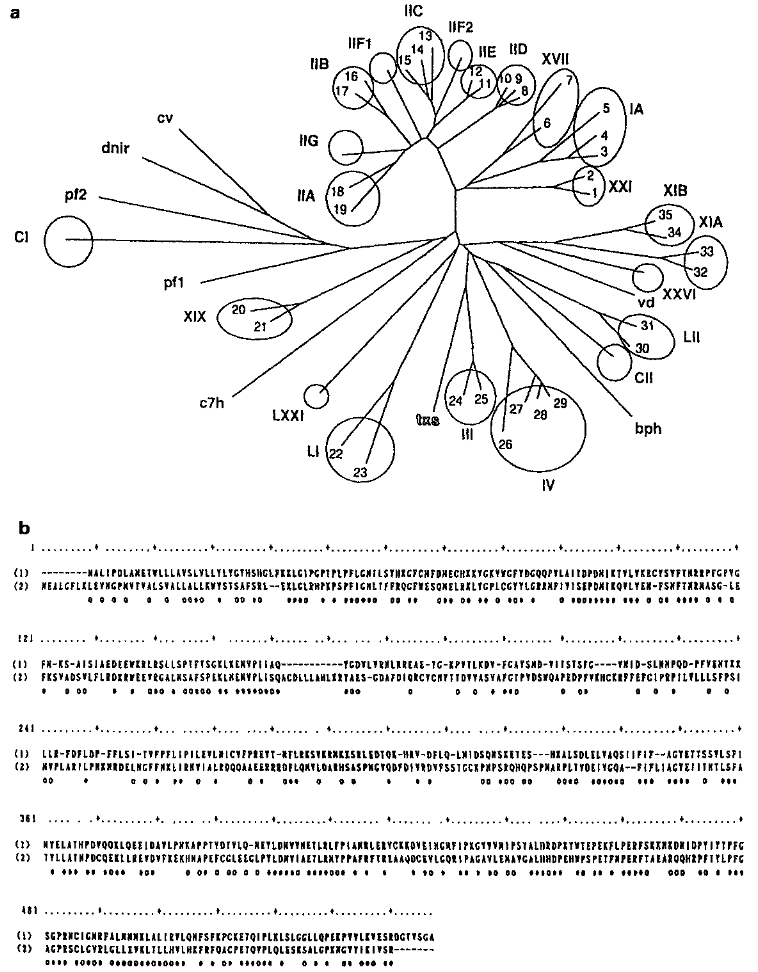

FIG. 4. (a) Unrooted tree of the cytochrome P-450 family including TXA synthase. Redrawn from the tree in Ref. 66. The nodes in a circle indicate proteins which belong to a group designated near the circle. Abbreviations are as follows: txs, human TXA synthase;[59] XXI 1, mouse P-450 XXI (A26660); XXI 2, human P-450 XXI (O4HUC2); IA 3, rabbit P-450 I (B27821); IA 4, mouse P-450 I (B23923); IA 5, rainbow trout P-450 I (A28789); XVII 6, chicken P-450 XVII (O4CHC7); XVII 7, bovine P-450 XVII (S04346); IID 8, human P-450 IID (S01199); IID 9, rat P-450 IID (S16874); IID 10, rat P-450 IID (D32970); IIE 11, human P-450 IIE (B25341); IIE 12, rat P-450 IIE (A25341); IIF2, chicken P-450 IIF2 (A31418); IIC 13, rat P-450 IIC (A34258); IIC 14, rabbit P-450 IIC (A22606); IIC 15, human P-450 IIC (S06306); IIF1, human P-450 IIF (A36036); IIB 16, rat P-450 IIB (A29818); IIB17, rabbit P-450 IIB (O4RBPB); IIG, rat P-450 IIG (A35551); IIA 18, human P-450 IIA (C34271); IIA 19, rat P-450 IIA (A31887); cv, *Streptomyces griseolus* P-450 CVA1 (A35401); dnir, *Fusarium oxysporum* P-450 dNIR (A40401); pf2, *Agrobacterium tumefaciens* pinF2 gene product (B32306); CI, *Pseudomonas putida* P-450 CI (A25660); pf1, *Agrobacterium tumefaciens* pinF1 gene product (A32306); XIX 20, chicken P-450 XIX (A31916); XIX 21, rat P-450 XIX (A36121); c7h, human cholesterol 7$\alpha$ hydroxylase;[104] LXXI, Avocado P-450 LXXI (A35867); LI 22, *Candida albicans* P-450 LI (S02713); LI 23, *Saccharomyces cerevisiae* P-450 LI (A27491); III 24, rabbit P-450 III (A29487); III 25, human P-450 III (A29815); IV 26, human P-450 IV (A33414); IV 27, rat P-450 IV (B32965); IV 28, rabbit P-450 IV (C34260); IV 29, rabbit P-450 IV (PG $\omega$-hydroxylase) (A29368); bph, *Aspergillus niger* benzoate-para-hydroxylase (S12015); CII, *Bacillus megaterium* P-450 CII (A34286); LII 30, *Candida maltosa* P-450 LII (A33254); LII 31, *Candida tropicalis* P-450 LII (JS0203); vd, rat 25-hydroxyvitamin $D_3$ 24-hydroxylase;[105] XXVI, rabbit P-450 XXVI (A33813); XIA 32, bovine P-450 XIA (O4BOM); XIA 33, rat P-450 XIA (A34164); XIB 34, rat P-450 XIB (A35342); XIB 35, bovine P-450 (A28415). The codes in parentheses indicate the accession numbers of the proteins in NBRF data base as references.

(b) Alignment between human TXA synthase[59] and human P-450 III, whose accession number in the NBRF database is A29815. Open circles, closed circles and '-' have the same meanings as those in Fig. 1(a).

twice during the course of molecular evolution. The tree also suggests that at least two types of plant lipoxygenases were generated by gene duplication before the divergence between soybean and garden pea.

### LTA Hydrolase

Leukotriene (LT) $A_4$ is synthesized from arachidonic acid by the activity of 5-lipoxygenase, and all kinds of LTs are synthesized via $LTA_4$. LTA

**a**

```

      1 .........+.........+.........+.........+.........+.........+.........+...

                  *    *    *                 *        *

(1)   AKCWVRNSDFQLHEIQYHLLNTHLVAEVIAVATMRCLPGLHPIFKFLIPHIRYTMEINTRARTQLISDGGIFD

(2)   AKCWVRSSDFQLHELHSHLLRGHLMAEVIAVATMRCLPSIHPIFKLLIPHFRYTMEINVRARNGLVSDLGIFD

(3)   AKCWVRSSDFQVHELNSHLLRGHLMAEVFTVATMRCLPSIHPVFKLIVPHLRYTLEINVRARNGLVSDFGIFD

(4)   AKCWVRSSDFQLHELQSHLLRGHLMAEVIVVATMRCLPSIHPIFKLIIPHLRYTLEINVRARTGLVSDMGIFD

(5)   AKIWVRSSDFHIHQTITHLLRTHLVSEVFGIAMYRQLPAVHPLFKLLVAHVRFTIAINTKAREQLNCEYGLFD

(6)   AKIWVRSSDFHVHQTITHLLRTHLVSEVFGIAMYRQLPAVHPIFKLLVAHVRFTIAINTKAREQLICECGLFD

(7)   SKAYVIVNDSCYHQLMSHWLNTHAAMEPFVIATHRHLSVLHPIYKLLTPHYRNNMNINALARQSLINANGIIE

(8)   AKAYVVVNDSCYHQLMSHWLNTHAVIEPFIIATNRHLSALHPIYKLLTPHYRDTMNINALARQSLINADGIIE

(9)   AKAYVVVNDSCYHQLVSHWLNTHAVVEPFIIATNRHLSVVHPIYKLLHPHYRDTMNINGLARLSLVNDGGVIE

(10)  AKAYVIVNDSCYHQLVSHWLNTHAVVEPFVIATNRHLSCLHPIYKLLYPHYRDTMNINSLARLSLVNDGGIIE

      o• o•  •   •o   • • o•   •   o•  • •o o••oo• o o• •  o ••  ••  •    •o o

```

**b**

```
      1 .........+.........+.........
         *    *    *    *
(1)   MCVFTCTAQHAAINQGQLDWYAWVPNAP
(2)   MCIFTCTGQHSSNHLGQLDWYTWVPNAP
(3)   MCIFTCTGQHSSIHLGQLDWFTWVPNAP
(4)   MCIFTCTGQHASVHLGQLDWYSWVPNAP
(5)   VVIFTASAQHAAVNFGQYDWCSWIPNAP
(6)   VVIFTASAQHAAVNFGQYDWCSWIPNAP
(7)   IIIWIASALHAAVNFGQYPYGGLIMNRP
(8)   IIIWTASALHAAVNFGQYPYGGFILNRP
(9)   IIIWTASALHAAVNFGQYPYGGLILNRP
(10)  IVIWTASALHAAVNFGQYSYGGLILNRP
      o oo  oo •oo   ••  o o o • •
```

**c**

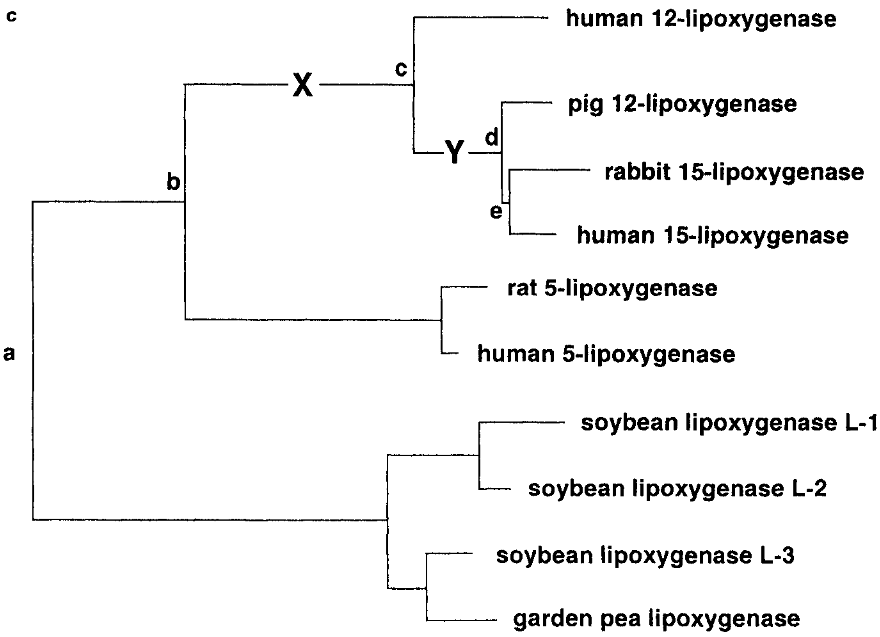

FIG. 5 (a) Alignment of a region including five invariant His residues of ten lipoxygenases. Redrawn from the alignment in Ref. 20. The invariant His residues are indicated by asterisks on the alignment. (1) Human 12-lipoxygenase,[72] (2) Pig 12-lipoxygenase,[74] (3) Rabbit 15-lipoxygenase,[76] (4) Human 15-lipoxygenase,[75] (5) Rat 5-lipoxygenase,[71] (6) Human 5-lipoxygenase,[69] (7) Soybean lipoxygenase L-1 (DASYL2), (8) Soybean lipoxygenase L-2 (DASYL1), (9) Soybean lipoxygenase L-3 (S01864), and (10) Garden pea lipoxygenase (S01142). The codes in the parentheses indicate the accession numbers of the proteins in the NBRF data base as references.

(b) Alignment of a region including a zinc finger-like motif, Cys-Cys-His-His, of lipoxygenases. Redrawn from the alignment in Ref. 20. The amino acid residues involved in the proposed motif are indicated by asterisks on the alignment. Open circles, closed circles, and '-' have the same meanings as those in Fig. 1(a). The numbers (1)–(10) correspond with those in Fig. 5(a).

(c) Phylogenetic tree of lipoxygenase family. Redrawn from the alignment in Ref. 20.

hydrolase is an enzyme which catalyses the hydrolysis of an epoxide moiety of $LTA_4$ to yield $LTB_4$. $LTB_4$ is a potent chemotactic compound for granulocytes and is closely related to the immune system.[80–82] The nucleotide sequences of cDNAs for this enzyme derived from human spleen, lung and placenta,[83,84] and mouse spleen[85] have been determined, all of which encode proteins 611 amino acid residues in length. The two human enzymes are identical to each other and the amino acid identity between human and mouse enzymes is 93%.

This enzyme shows weak but significant sequence homology to the members of the aminopeptidase N family (Fig. 6(a)),[86,87] although the functions of LTA hydrolase is quite different from that of aminopeptidases. Figure 6(b) shows the phylogenetic relationships within the aminopeptidase family including LTA hydrolases.[87] One of the highly conserved regions between them corresponds with a ubiquitous sequence motif found in a wide variety of zinc metalloenzymes.[87,88] The motif sequence, VXXHEXXH (alignment sites 349–356), contains two invariant His residues, which act as ligands for the zinc ion in the proteases. In addition, it is known that an invariant Glu residue in the motif is one of the active sites of the peptidases. These residues are also conserved in the LTA hydrolase.

After the determination of the sequence homology, it was revealed by atomic absorption spectrometry that LTA hydrolase contains one zinc ion per enzyme molecule.[89,90] Furthermore, LTA hydrolase exhibits peptidase activity toward the synthetic substrates alanine-4-nitroanilide and leucine-4-nitroanilide.[89,91] The zinc ion was shown to be involved in both LTA hydrolase activity and peptidase activity of the enzyme, that is, the apoenzyme of LTA hydrolase is virtually inactive for both activities but can be reactivated to show both activities by the addition of stoichiometric amounts of zinc or cobalt ion. The LTA hydrolase undergoes suicide type inactivation, and the inactivated LTA hydrolase does not show peptidase activity. The experimental results, in addition to the

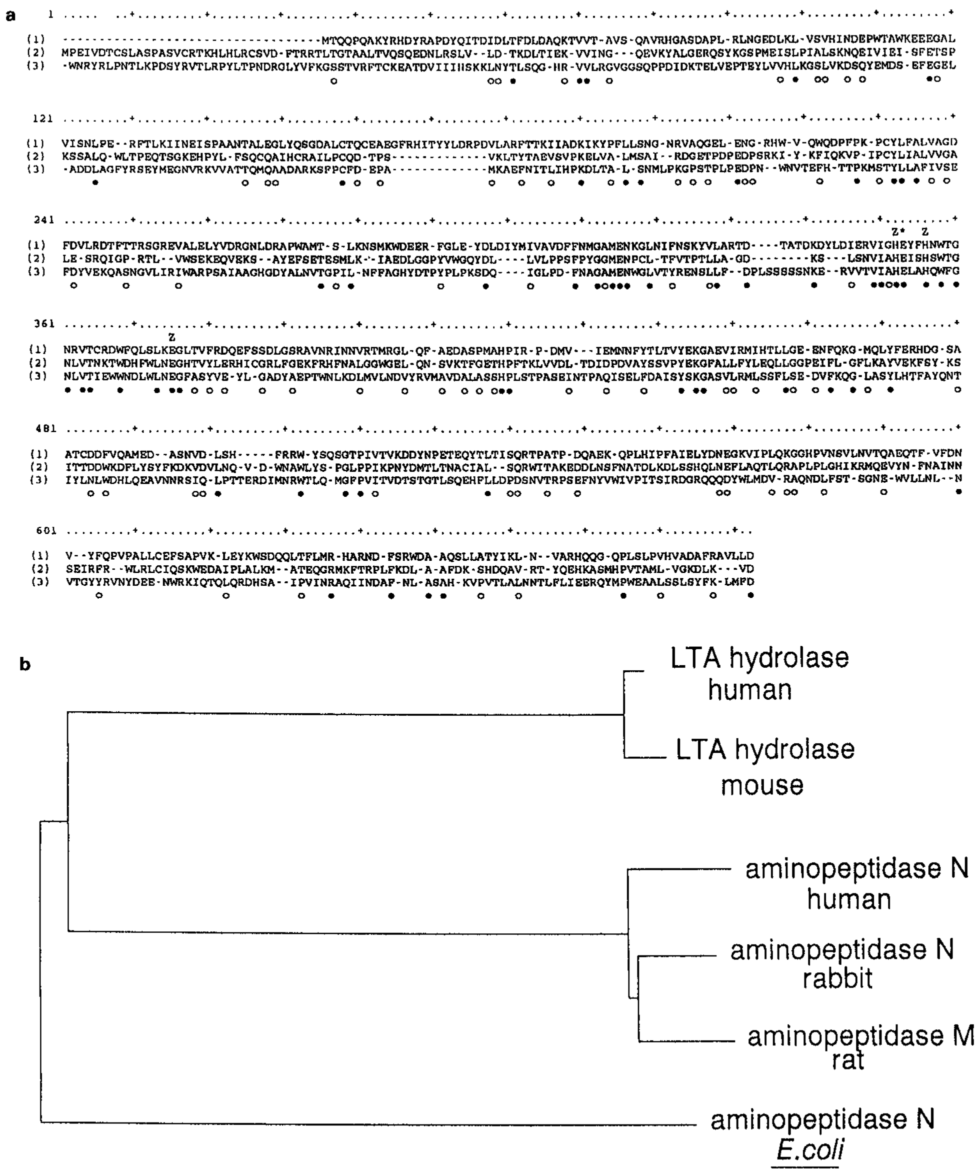

FIG. 6 (a) Alignment of aminopeptidase N family including LTA hydrolases. Redrawn from the alignment in Ref. 87. Open circles, closed circles, and '-' have the same meanings as those in Fig. 1(a). 'Z' indicates the site which is considered to act as a ligand for zinc ion, while '*' indicates the site which is considered to be an active site for peptidase activity. (1) *E. coli* aminopeptidase N (DPECN), (2) Human LTA hydrolase,[84] and (3) Human aminopeptidase N (S01658). The codes in the parentheses indicate the accession numbers of the proteins in the NBRF data base as references.

(b) Phylogenetic tree of aminopeptidase N family. Redrawn from the alignment in Ref. 87. The references or accession numbers for human LTA hydrolase, human aminopeptidase N, and *E. coli* aminopeptidase N are the same as those in Fig. 6(a). The accession number for rabbit aminopeptidase N in the NBRF data base is S07099. The references for mouse LTA hydrolase and rat aminopeptidase M are Ref. 85 and 86, respectively.

sequence homology, suggest that the catalytic mechanism of LTA hydrolase is similar to those of zinc metallopeptidases.

It has been a riddle that LTA hydrolase activity is found in a wide variety of tissues and cells, some of which do not express 5-lipoxygenase activity and, therefore, are unable to synthesize LTA, the substrate for this enzyme. The finding of peptidase activity of this enzyme offers an interpretation that this enzyme may exert different functions in different cells and tissues.[91] Further discussion should be suspended until peptidase activity of this enzyme is found *in vivo*. However, it should be noted that LTA hydrolase is a new type of enzyme which can utilize two different cellular compounds, lipids and peptides, as substrates.

### Conclusion

We have discussed the structure, function and molecular evolution of the enzymes involved in eicosanoid synthesis. Each enzyme discussed here has a different origin from another, and sometimes shows unusual strategies for molecular evolution (acquisition of enzymatic activity by PGD synthase, gene sharing by PGF synthase, utilization of two different types of substrates by LTA hydrolase). One of our goals is to describe the evolution of biochemical pathways for eicosanoid metabolism. However, the sequence data available now are too scanty for us to make any definite description about the evolution of the arachidonic acid cascade. Further, experimental and theoretical approaches will reveal not only the evolution of the enzymes involved in the arachidonic acid cascade, but also the evolution of the cascade itself.

### References

1. Ito S, Narumiya S, Hayaishi O. *Prostaglandins, Leukotrienes and Essent Fatty Acids* 1989; **37**: 219–234.
2. Yamamoto S. *Prostaglandins, Leukotrienes Essent Fatty Acids* 1989; **35**: 219–229.
3. Shimizu T, Wolfe LS. *J Neurochem* 1990; **55**: 1–15.
4. Sigal E. *Am J Physiol* 1991; **260**: L13–L28.
5. Hirata M, Hayashi Y, Ushikubi F, *et al. Nature* 1991; **349**: 617–620.
6. Dixon RAF, Diehl RE, Opas E, *et al. Nature* 1990; **343**: 282–284.
7. Miyamoto T, Ogino N, Yamamoto S, Hayaishi O. *J Biol Chem* 1976; **251**: 2629–2636.
8. Ohki S, Ogino N, Yamamoto S, Hayaishi O. *J Biol Chem* 1979; **254**: 829–836.
9. Ogino N, Ohki S, Yamamoto S, Hayaishi O. *J Biol Chem* 1978; **253**: 5061–5068.
10. van der Ouderaa FJ, Buytenhek M, Nugteren DH, van Dorp DA. *Biochim Biophys Acta* 1977; **487**: 315–331.
11. Yokoyama C, Takai T, Tanabe T. *FEBS Lett* 1988; **231**: 347–351.
12. Merlie JP, Fagan D, Mudd J, Needleman P. *J Biol Chem* 1988; **263**: 3550–3553.
13. DeWitt DL, Smith WL. *Proc Natl Acad Sci USA* 1988; **85**: 1412–1416, and correction p. 5056.
14. DeWitt DL, El-Harith EA, Kraemer SA, *et al. J Biol Chem* 1990; **265**: 5192–5198.
15. Takahashi Y, Ueda N, Yoshimoto T, *et al. Biochem Biophys Res Comm* 1992; **182**: 433–438.
16. Yokoyama C, Tanabe T. *Biochem Biophys Res Comm* 1989; **165**: 888–894.
17. Xie W, Chipman JG, Robertson DL, Erikson RL, Simmons DL. *Proc Natl Acad Sci USA* 1991; **88**: 2692–2696.
18. Kujubu DA, Fletcher BS, Varnum BC, Lim RW, Herschman HR. *J Biol Chem* 1991; **266**: 12866–12872.
19. Fletcher BS, Kujubu DA, Perrin DM, Herschman HR *J Biol Chem* 1992; **267**: 4338–4344.
20. Toh H, Yokoyama C, Tanabe T, Yoshimoto T, Yamamoto S. *Prostaglandins* 1992; in press.
21. Toh H. *FEBS Lett* 1989; **258**: 317–319.
22. Appella E, Weber IT, Blasi F. *FEBS Lett* 1988; **231**: 1–4.
23. Doolittle RF, Feng DF, Johnson MS, McClure MA. *Cold Spring Harbor Symp Quant Biol* 1986; **51**: 447–455.
24. Rollins TE, Smith WL. *J Biol Chem* 1980; **255**: 4872–4875.
25. Libert F, Ruel J, Ludgate M, *et al. EMBO J* 1987; **6**: 4193–4196.
26. Ueno R, Narumiya S, Ogorochi T, Nakayama T, Hayaishi O. *Proc Natl Acad Sci USA* 1982; **79**: 6093–6097.
27. Hayaishi O. *J Biol Chem* 1988; **263**: 14593–14596.
28. Urade Y, Nagata A, Suzuki Y, Fujii Y, Hayaishi O. *J Biol Chem* 1989; **264**: 1041–1045.
29. Nagata A, Suzuki Y, Igarashi M, *et al. Proc Natl Acad Sci USA* 1991; **88**: 4020–4024.
30. Igarashi M, Nagata A, Toh H, Urade Y, Hayaishi O. *Proc Natl Acad Sci USA* 1992; **89**: 5376–5380.
31. Peitsch MC, Boguski MS. *TIBS* 1991; **16**: 363.
32. Ali S, Clark AJ. *J Mol Biol* 1988; **199**: 415–426.
33. Pervaiz S, Brew K. *FASEB J* 1987; **1**: 209–214.
34. Ptitsyn OB, Finkelstein AV. *Quart Rev Biophys* 1980; **13**: 339–386.
35. Branden CI. *Quart Rev Biophys* 1980; **13**: 317–338.
36. Richardson JS. *Advan Protein Chem* 1981; **34**: 167–339.
37. Papiz MZ, Sawyer L, Eliopoulos EE, *et al. Nature* 1986; **324**: 383–385.
38. Monaco HL, Zanotti G, Spadon P, Bolognesi M, Sawyer L, Eliopoulos EE. *J Mol Biol* 1987; **197**: 695–706.
39. Newcomer ME, Jones TA, Åqvist J, *et al. EMBO J* 1984; **3**: 1451–1454.
40. Cowan SW, Newcomer ME, Jones TA. *PROTEINS* 1990; **8**: 44–61.
41. Holden HM, Rypniewski WR, Law JH, Rayment I. *EMBO J* 1987; **6**: 1565–1570.
42. Huber R, Schneider M, Epp O, *et al. J Mol Biol* 1987; **195**: 423–434.
43. Spence AM, Sheppard PC, Davie JR, *et al. Proc Natl Acad Sci USA* 1989; **86**: 7843–7847.
44. Watanabe K, Yoshida R, Shimizu T, Hayaishi O. *J Biol Chem* 1985; **260**: 7035–7041.
45. Watanabe K, Iguchi Y, Iguchi S, Arai Y, Hayaishi O, Roberts LJ (II). *Proc Natl Acad Sci USA* 1986; **83**: 1583–1587.
46. Watanabe K, Fujii Y, Nakayama K, *et al. Proc Natl Acad Sci USA* 1988; **85**: 11–15.
47. Kuchinke W, Barski O, Watanabe K, Hayaishi O. Biochem Biophys Res Commun 1992; **183**: 1238–1246.
48. Hayaishi O, Watanabe K, Fujii Y, *et al.* Prostaglandin F synthase, a dual function enzyme. In: King TE, Mason HS, Morrison M, eds. *Progress in Clinical and Biological Research, Vol. 274, Oxidases and Related Redox Systems.* Alan R. Liss, Inc.: New York, 1988; 577–587.
49. Hayashi H, Fujii Y, Watanabe K, Urade Y, Hayaishi O. *J Biol Chem* 1989; **264**: 1036–1040.
50. Urade Y, Watanabe K, Eguchi N, Fujii Y, Hayaishi O. *J Biol Chem* 1990; **265**: 12029–12035.
51. Fujii Y, Watanabe K, Hayashi H, *et al. J Biol Chem* 1990; **265**: 9914–9923.
52. Piatigorsky J, Wistow GJ. *Cell* 1989; **57**: 197–199.
53. Rondeau JM, Tête-Favier F, Podjarny A, *et al. Nature* 1992; **355**: 469–472.
54. Needleman P, Turk J, Jakschik BA, Morrison AR, Lefkowith JB. *Ann Rev Biochem* 1986; **55**: 69–102.
55. Ullrich V, Nüsing R. *Stroke* 1990; **21** (suppl IV): IV-134–IV-138.
56. Ogletree ML. *Fed Proc* 1987; **46**: 133–138.
57. DeWitt DL, Smith WL. *J Biol Chem* 1983; **258**: 3285–3293.
58. Nüsing R, Schneider-Voss S, Ullrich V. *Arch Biochem Biophys* 1990; **280**: 325–330.
59. Yokoyama C, Miyata A, Ihara H, Ullrich V, Tanabe T. *Biochem Biophys Res Comm* 1991; **178**: 1479–1484.
60. Ohashi K, Ruan K-H, Kulmacz RJ, Wu K K, Wang L-H. *J Biol Chem* 1992; **267**: 789–793.
61. Nebert DW, Gonzalez FJ. *Ann Rev Biochem* 1987; **56**: 945–993.
62. Nebert DW, Nelson DR, Adesnik M, *et al. DNA* 1989; **8**: 1–14.
63. Gonzalez FJ. *Pharmacol Rev* 1989; **40**: 243–275.
64. McGiff JC *Ann Rev Pharmacol Toxicol* 1991; **31**: 339–369.
65. Matsubara S, Yamamoto S, Logawa K, *et al. J Biol Chem* 1987; **262**: 13366–13371.
66. Miyata A, Yokoyama C, Toh H, Tanabe T. Unpublished.
67. Poulos TL, Finzel BC, Howard AJ. *J Mol Biol* 1987; **195**: 687–700.
68. Zvelebil MJJM, Wolfe CR, Sternberg MJE. *Protein Engng* 1991; **4**: 271–282.
69. Dixon RAF, Jones RE, Diehl RE, Bennett CD, Kargman S, Rouzer CA. *Proc Natl Acad Sci USA* 1988; **85**: 416–420.
70. Matsumoto T, Funk CD, Rådmark O, Höög J-O, Jörnvall H, Samuelsson B. *Proc Natl Acad Sci USA* 1988; **85**: 26–30.
71. Balcarek JM, Theisen TW, Cook MN, *et al. J Biol Chem* 1988; **263**: 13937–13941.
72. Yoshimoto T, Yamamoto Y, Arakawa T, *et al. Biochem Biophys Res Commun* 1990; **172**: 1230–1235.
73. Funk CD, Furci L, FitzGerald GA. *Proc Natl Acad Sci USA* 1990; **87**: 5638–5642.
74. Yoshimoto T, Suzuki H, Yamamoto S, Takai T, Yokoyama C, Tanabe T. *Proc Natl Acad Sci USA* 1990; **87**: 2142–2146.
75. Sigal E, Craik CS, Highland E, *et al. Biochem Biophys Res Comm* 1988; **157**: 457–464.
76. Fleming J, Thiele BJ, Chester J, *et al. Gene* 1989; **79**: 181–188.
77. Funk CD, Hoshiko S, Matsumoto T, Rådmark O, Samuelsson B. *Proc Natl Acad Sci USA* 1989; **86**: 2587–2591.
78. O'Prey J, Chester J, Thiele BJ, *et al. Gene* 1989; **84**: 493–499.
79. Shibata D, Steczko J, Dixon JE, Andrews PC, Hermodson M, Axelrod B. *J Biol Chem* 1988; **263**: 6816–6821.
80. Samuelsson B, Dahlén S-E, Lindgren JÅ, Rouzer CA, Serhan CN. *Science* 1987; **237**: 1171–1176.
81. Shimizu T. *Int J Biochem* 1988; **20**: 661–666.
82. Samuelsson B, Funk CD. *J Biol Chem* 1989; **264**: 19469–19472.
83. Funk CD, Rådmark O, Fu JY, *et al. Proc Natl Acad Sci USA* 1987; **84**: 6677–6681.
84. Minami M, Ohno S, Kawasaki H, *et al. J Biol Chem* 1987; **262**: 13873–13876.
85. Medina JF, Rådmark O, Funk CD, Haeggström JZ. *Biochem Biophys Res Comm* 1991; **176**: 1516–1524.
86. Malfroy B, Kado-Fong H, Gros C, Giros B, Schwartz J-C, Hellmis R. *Biochem Biophys Res Comm* 1989; **161**: 236–241.
87. Toh H, Minami M, Shimizu T. *Biochem Biophys Res Comm* 1990; **171**: 216–221.
88. Vallee BL, Auld DS. *Biochemistry* 1990; **29**: 5647–5659.
89. Minami M, Ohishi N, Mutoh H, *et al. Biochem Biophys Res Comm* 1990; **173**: 620–626.
90. Haeggström JZ, Wetterholm A, Shapiro R, Vallee BL, Samuelsson B. *Biochem Biophys Res Comm* 1990; **172**: 965–970.
91. Haeggström JZ, Wetterholm A, Vallee BL, Samuelsson B. *Biochem Biophys Res Comm* 1990; **173**: 431–437.
92. Venturelli D, Bittenbender S, Rovera G. *Nucleic Acids Res* 1989; **17**: 7987–7988.
93. Morishita K, Tsuchiya M, Asano S, Kaziro Y, Nagata S. *J Biol Chem* 1987; **262**: 15208–15213.

94. Clark AJ, Clissold PM, Al Shawi R, Beatie P, Bishop J. *EMBO J* 1984; **3**: 1045–1052.
95. Vetr H, Gebhard W. *Biol Chem Hoppe-Seyler* 1990; **371**: 1185–1196.
96. Dente L, Pizza MG, Metspalu A, Cortese R. *EMBO J* 1987; **6**: 2289–2296.
97. Ichiyoshi Y, Endo H, Yamamoto M. *Biochim Biophys Acta* 1987; **910**: 45–51.
98. Neil Dear T, Campbell K, Rabbitts TH. *Biochemistry* 1991; **30**: 10376–10382.
99. Keen JN, Caceres I, Eliopoulos EE, Zagalsky PF, Findlay JBC *Eur J Biochem* 1991; **197**: 407–417.
100. Zagalsky PF, Eliopoulos EE, Findlay JBC. *Comp Biochem Physiol* 1990; **97B**: 1–18.
101. Anderson S, Marks CB, Lazarus R, *et al. Science* 1985; **230**: 144–149.
102. Oechsner U, Magdolen V, Bandlow W. *FEBS Lett* 1988; **238**: 123–128.
103. Wermuth B, Omar A, Forster A, *et al. Prog Clin Biol Res* 1987; **232**: 297–307.
104. Noshiro M, Okuda K. *FEBS Lett* 1990; **268**: 137–140.
105. Ohyama Y, Noshiro M, Okuda K. *FEBS Lett* 1991; **278**: 195–198.

### Note added in proof:

Recently, the crystal structure of myeloperoxidase was determined (Zengl, Fenna RE. *J Mol Biol* 1992; **226**: 185–207), which may be useful in investigations of the structure and function of cyclooxygenase.

**Received 1 May 1992; accepted 20 May 1992**
